# Analysis of E2 gene integrity in HPV16 and HPV58 viruses isolated from women with cervical pathology

**DOI:** 10.1590/0074-02760160269

**Published:** 2016-10-31

**Authors:** María del R González-Losa, Marylin Puerto-Solis, Juan Tenorio Ruiz, Ariel I Rosado-López, Oscar Hau-Aviles, Guadalupe Ayora-Talavera, Isidro Cisneros-Cutz, Laura Conde-Ferráez

**Affiliations:** 1 Universidad Autónoma de Yucatán, Centro de Investigaciones Regionales Dr Hideyo Noguchi, Laboratorio de Virología, Mérida, Yucatán, México; 2 Clínica de Colposcopía, Hospital General O’Horán, Mérida, Yucatán, México; 3 Clínica de Colposcopía, Hospital General Valladolid, Valladolid, Yucatán, México

**Keywords:** HPV58, HPV16, integration, E2

## Abstract

Integration of human papillomavirus (HPV) DNA into human cells accompanied by the disruption of the viral genome has been described as a prerequisite for cancer development. This study aimed to investigate E2 gene integrity of HPV16 and HPV58 viruses isolated from infected women with cervical lesions. Forty-two HPV16- and 31 HPV58-positive samples were analysed. E2 integrity was assumed when all fragments covering the E2 gene were amplified with specific polymerase chain reaction primers. Overall, in 59% of the samples, at least one fragment was not amplified in HPV16- (57%) and HPV58-positive samples (61%). Samples from high-grade squamous intraepithelial lesions had the highest frequency of E2 gene disruptions (73%), followed by samples from low-grade squamous intraepithelial lesions (63%) and, finally, samples from invasive cervical cancer (35%). Association between the integrity status of the E2 gene, and lesion grade was assessed by the chi-squared test applied to the combined set of viruses (p = 0.6555) or to populations of the same virus type (HPV58, p = 0.3101; HPV16, p = 0.3024). In conclusion, in this study, no association was found between the presence of E2 gene disruptions and the grade of cervical lesions caused by HPV16 and HPV58.

The oncogenic role of human papillomavirus (HPV) has been established through decades of biological and epidemiological studies. HPV is responsible for 5.2% of all neoplasias worldwide (Parkin 2006). The relationship between cervical cancer (CC) and HPV is well known, because HPV infection increases the risk of CC development (Walboomers et al. 1999).

Worldwide, in healthy women with normal cervical cytology, HPV is most prevalent (30%) in women under 25 years of age. In Africa and Oceania, these rates are even higher: 45% and 50%, respectively (Bruni et al. 2016). In 2012, 527,600 new cases of CC were diagnosed worldwide. In Americas, 83,200 cases were documented with significant variations between different regions. More than half of all cases (45,000) were from South America (Ferlay et al. 2015). In Mexico, 2,738 new cases were reported in 2015 (SS 2016).

HPV16 and HPV58 types, associated with high-grade squamous intraepithelial lesions (HSILs) and CC, are classified as “oncogenic or high risk viruses”. Worldwide, HPV16 is the most frequent type of HPV, whereas HPV58 is the seventh most prevalent type (Li et al. 2011). However, geographical variations in the distribution of these HPV types have been reported (Chan et al. 1999, Herrero et al. 2000). For example, HPV58 is as frequent as HPV16 in women with cervical lesions living in Southern Mexico (González-Losa & Conde-Ferraez 2013).

HPV has a circular double stranded DNA genome that can be present in the infected cell as an extrachromosomal molecule (episomal state), an integrated element within the host genome, or a combination of these two states. Collectively, such states determine the viral genome status (Vojtechova et al. 2016). During the episomal state, the E2 protein is responsible for down-regulating the transcription of the E6 and E7 oncogenes with a low risk to develop cancer. The first step in the viral integration is the linearisation of circular DNA that can disrupt the E2 open reading frame, leading to the loss of the E2 protein and, as a result, increased expression of the E6 and E7 proteins (Ho et al. 2011).

Because HPV genome integration is necessary for malignant transformation, the assessment of E2 gene integrity is useful for the determination of the viral genome status. The aim of this study was to assess E2 gene integrity in HPV16- and HPV58-positive samples as an indicator of the integration of the HPV genome into the host DNA and to determine a possible association of the viral genome status with the grade of cervical lesion.

This cross-sectional study was performed by using samples from women who attended the Colposcopy Clinic of the General Hospital in Valladolid and the O’Horán General Hospital in Merida (both located in Yucatan, Mexico). These establishments are public hospitals of the Mexican Ministry of Health that provide medical service to women without social security insurance. All patients were referred to the colposcopy service after having an abnormal Pap smear during a routine check-up. During the colposcopy procedure, women were invited to participate in the project. After signing an informed consent document, cervical samples were obtained with a cytobrush and placed into 50% ethanol at room temperature until laboratory processing. In order to corroborate cytological and colposcopy diagnosis, all women had a biopsy. This protocol was reviewed and approved by the Bioethics Board of the O’Horán General Hospital (CIE-044-2-11).

DNA extraction was performed by experienced personnel using the DNeasy Blood and Tissue Kit (QIAGEN) according to the manufacturer’s instructions. For DNA quality control, β-globin amplification was performed by using PC04 and GH20 primers (260-bp amplicon) (Saiki et al. 1986). Nested polymerase chain reaction (PCR) was used to detect HPV16 and HPV58 following the amplification of the E6 and E7 gene fragments by a previously reported methodology (Sotlar et al. 2004). Clinical samples identified by the same method and by sequencing procedures served as positive controls. As a negative control, we used water instead of DNA solution.

To evaluate the integrity of the E2 gene in HPV16-positive samples, three pairs of primers were used in separate reactions according to the methodology reported by Li et al. (2008). The amplicons were overlapping fragments covering the E2 gene as follows: amino-terminus (457 bp), hinge (477 bp) and carboxy-terminus (276 bp). DNA from SiHa cells, kindly donated by Dr Marcela Lizano Soberon, was used as positive control of the disrupted HPV16 E2 gene.

To evaluate the integrity of the E2 gene in HPV58-positive samples, four pairs of primers were used in separate reactions according to the methodology reported previously (Chan et al. 2007). The amplicons were overlapping fragments covering the E2 gene as follows: amino-terminus 1 (182 bp), amino-terminus 2 (192 bp); hinge (199 bp) and carboxy-terminus (113 bp).

In total, 73 specimens were analysed: 42 HPV16-positive samples (57.5%) and 31 HPV58-positive samples (42.5%). With respect to categorising diagnoses by the histopathological features, 44% of the samples were obtained from low-grade squamous intraepithelial lesions (LSILs), 34% were associated with HSILs and 22% came from women with CC. The whole E2 gene was detected in 41% of the samples, whereas in 59% of the samples, the E2 gene was disrupted, because at least one fragment was not amplified. With regard to the relationship between the histopathological diagnosis and the physical status of the viral genome, HSIL samples had the highest frequency of E2 gene disruptions (73%), whereas E2 was disrupted only in 63% of LSIL samples. In invasive CC, E2 gene disruption was found in 35%.

Twenty-one percent of HPV16-positive samples came from women with LSIL, whereas 50% and 29% were associated with HSIL and CC, respectively. With regard to E2 gene integrity, the whole gene could be amplified in 43% of HPV16-positive samples. In the remaining 14% and 43% of HPV16-positive samples, either one or two segments were not amplified, respectively. The carboxy-terminus was the most frequently lost segment, whereas the hinge was the most conserved region.

Out of 31 HPV58-positive women; 70% presented with LSIL; 17% had HSIL and 10% were diagnosed with CC. For one person (3%), diagnosis information was missing. The whole E2 gene was amplified in 39% of HPV58-positive samples, whereas in 58% of the samples, at least one fragment could not be amplified. Moreover, in one case (3%), no segments could be amplified at all. The amino-terminus 2 was the most frequently disrupted segment (55%), followed by the hinge (36%), the carboxy-terminus (23%) and the amino-terminus 1 (2%). Figure illustrates the relationships between E2 gene integrity on one hand and histopathological diagnosis and type of the virus on the other hand.

**Figure:**
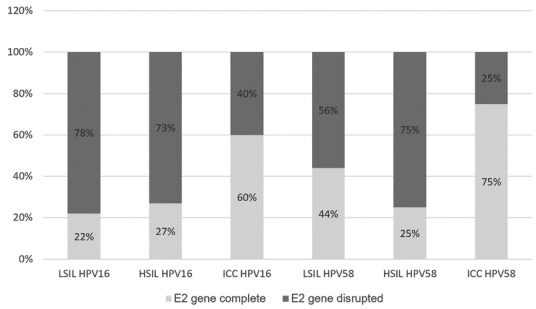
HSILs: high grade squamous intraepithelial lesions; ICC: invasive cervical cancer; LSILs: low grade squamous intraepithelial lesions.

In order to determine potential associations between the integrity of the E2 gene and lesion grade, the chi squared tests were performed. All HPV-positive cases were divided into LSIL-, HSIL- and CC-associated cases were in for the analyses. No significant associations between pathological manifestation and E2 gene integrity were found when both virus types were combined (p = 0.6555) or considered individually (HPV58, p = 0.3101; HPV16, p = 0.3024).

Viral DNA integration into the host genome is a crucial event for cancer development. Therefore, viral DNA integrity has been proposed, albeit controversially (Chan et al. 2007, Cricca et al. 2009, Manawapat et al. 2012), as a possible biomarker for the cancer risk, mainly in cases of infection with HPV16 and/or HPV18 (Oliveira et al. 2013, Tsakogiannis et al. 2015, Xu et al. 2015). In addition, HPV58 viruses have been found to be very frequent in Mexican women with cervical pathology living in the Mayan area (González-Losa & Conde-Ferraez 2013). Our results reinforce these observations and reveal that the frequency of E2 gene disruptions in the HPV58 genome is higher than that in the genome of HPV16.

The analysis of the obtained results reveals several findings common for both genotypes. (i) < 50% of women with LSIL have an incomplete E2 gene; (ii) the percentage of HSIL samples that exhibit integrated viral genome is similar in cases of HPV16 and HPV58 infections; (iii) contrary to the generally held view, CC samples positive for either HPV16 or HPV58 rarely express an incomplete E2 gene.

We found that the pattern of the E2 gene disruptions was different in HPV16- and HPV58-positive samples. The region most frequently disrupted in HPV16-positive samples was the carboxy-terminus, whereas for HPV58-positive samples, it was the amino-terminus 2. The biological significance of this observation is unknown.

Incomplete viral genome was detected with the highest frequency in HSIL samples and at a similar rate for both genotypes. The frequency of cases with complete HPV16 genome integration in HSIL lesions has been reported to range from 36% to 100%, depending on the study population (Andersson et al. 2005, Li et al. 2008). Little information about prevalence of HPV58 genome integration is available. In studies of samples obtained from Chinese women, Chan et al. (2007) reported results that were similar to ours. Collectively, these results are in agreement with known natural history of CC.

The frequency of complete E2 gene integration in CC samples was higher than expected. This can be explained by the existence of the viral genome in both episomal and integrated states. Although this phenomenon was not evaluated in our study, it has been previously reported for HPV16 (Peitsaro et al. 2002, Cricca et al. 2009). Moreover, Manawapat et al. (2012) reported the occurrence of both forms of the viral genome in 92% of women without cervical lesions and persistent infection, as well as in 90% of women with HSIL.

Recent evidence shows that all viral genes can be broken during the integration process, including the late and long control region. In fact, in the study of HPV16 viruses by Hu et al. (2015), it was found that E1, L1 and L2 genes were disrupted more often than E2. Those results do not invalidate our work but suggest that it patients have a complete E2 gene, it does not necessarily mean that the whole HPV genome is in the episomal state, because other, non-analysed genes may be disrupted.

In conclusion, in our study we found that there was no association between the presence of HPV E2 gene disruption and the grade of cervical lesions. Therefore, the presence of the disrupted E2 gene cannot be considered as a marker of cervical pathology.

## References

[B1] Andersson S, Safari H, Mints M, Lewensohn-Fuchs I, Gyllensten U, Johansson B (2005). Type distribution, viral load and integration status of high-risk
human papillomaviruses in pre-stages of cervical cancer (CIN). Br J Cancer.

[B2] Bruni L, Barrionuevo-Rosas L, Albero G, Aldea M, Serrano B, Valencia S, ICO Information Centre on HPV and Cancer (HPV Information Centre) (2016). Human papillomavirus and related diseases in the world.

[B3] Chan PK, Cheung JLK, Cheung TH, Lo KWK, Yim SF, Siiu SSN (2007). Profile of viral load, integration and E2 gene disruption of HPV58 in
normal cervix and cervical neoplasia. J Infect Dis.

[B4] Chan PKS, Li WH, Chan MYM, Ma WL, Cheung LK, Cheng AF (1999). High prevalence of human papillomavirus type 58 in Chinese women with
cervical cancer and precancerous lesions. J Med Virol.

[B5] Cricca M, Venturoli V, Leoa E, Costa S, Musiani M, Zerbini M (2009). Disruption of HPV 16 E1 and E2 genes in precancerous cervical
lesions. J Virol Methods.

[B6] Ferlay J, Soerjomataram I, Dikshit R, Ese S, Mathers C, Rebelo M (2015). Cancer incidence and mortality worldwide: sources, methods and major
patterns in GLOBOCAN 2012. Int J Cancer.

[B7] González-Losa MR, Conde-Ferraez L, Smith HB (2013). Prevalence and distribution of HPV 16, 18 and 58 in
southeast Mexico. Handbook of human papillomavirus. Prevalence, detection and
management.

[B8] Herrero R, Hidelsheim A, Bratti C, Sherman M, Hutchinson M, Morales J (2000). Population-based study of human papillomavirus infection and cervical
neoplasia in rural Costa Rica. J Natl Cancer Inst.

[B9] Ho CHM, Lee BH, Changf SF, Chiena TY, Huangb SH, Yane CC (2011). Integration of human papillomavirus correlates with high levels of
viral oncogene transcripts in cervical carcinogenesis. Virus Res.

[B10] Hu Z, Zhu D, Wang W, Li W, Jia W, Zeng X (2015). Genome-wide profiling of HPV-integration in cervical cancer identifies
clustered genomic hoy spots and a potential microhomology-mediated integration
mechanism. Nat Genet.

[B11] Li N, Franceschi S, Howell-Jones R, Snijders PJF, Clifford GM (2011). Human papillomavirus type distribution in 30,848 invasive cervical
cancers worldwide: variation by geographical region, histological type and year of
publication. Int J Cancer.

[B12] Li W, Wang W, Si M, Han L, Gao Q, Luo A (2008). The physical state of HPV 16 infection and its clinical significance
in cancer precursor lesion and cervical carcinoma. J Cancer Res Clin Oncol.

[B13] Manawapat A, Stubenrauch F, Russ R, Munk C, Kjær SK, Iftner T (2012). Physical state and viral load as predictive biomarkers for persistence
and progression of HPV16-positive cervical lesions: results from a population
based long-term prospective cohort study. Am J Cancer Res.

[B14] Oliveira G, Delgado C, Verdasca N, Pista A (2013). Prognostic value of human papillomavirus types 16 and 18 DNA physical
status in cervical intraepithelial neoplasia. Clin Microbiol Infect.

[B15] Parkin DM (2006). The global health burden of infection-associated cancer in the year
2002. Int J Cancer.

[B16] Peitsaro P, Johansson B, Syrjänen S (2002). Integrated human papillomavirus type 16 is frequently found in
cervical cancer precursors as demonstrated by a novel quantitative real-time PCR
technique. J Clin Micriobiol.

[B17] Saiki RK, Bugawan TL, Horn GT, Mullis KB, Erlich HA (1986). Analysis of enzymatically amplified beta-globin and HLA-DQ alpha DNA
with allele-specific oligonucleotide probes. Nature.

[B18] Sotlar K, Diemer D, Dethleffs A, Hack Y, Stubner A, Vollmer N (2004). Detection and typing of human papillomavirus by E6 nested multiplex
PCR. J Clin Microbiol.

[B19] SS - Secretaria de Salud Boletín epidemiológico.

[B20] Tsakogiannis D, Gortsilas P, Kyriakopoulou Z, Ruether IGA, Dimitriou TG, Orfanoudakis G (2015). Sites of disruption within E1 and E2 genes of HPV16 and association
with cervical dysplasia. J Med Virol.

[B21] Vojtechova Z, Sabol I, Salakova M, Turek L, Grega M, Smahelova J (2016). Analysis of the integration of human papillomaviruses in head and neck
tumours in relation to patients prognosis. Int J Cancer.

[B22] Walboomers J, Jacobs M, Manos MM, Bosch FX, Kummer JA, Shah KV (1999). Human papillomavirus is a necessary cause of invasive cervical cancer
worldwide. J Pathol.

[B23] Xu F, Cao M, Shi Q, Chen H, Wang Y, Li X (2015). Integration of the full-length HPV 16 genome in cervical cancer and
Caski and Siha cell lines and the possible ways of HPV integration. Virus Genes.

